# Assembly of metallic nanoparticle arrays on glass via nanoimprinting and thin-film dewetting

**DOI:** 10.3762/bjnano.8.106

**Published:** 2017-05-12

**Authors:** Sun-Kyu Lee, Sori Hwang, Yoon-Kee Kim, Yong-Jun Oh

**Affiliations:** 1 Department of Advanced Materials Science and Engineering, Hanbat National University, 125 Dongseo-daero, Yuseong-gu, Daejeon 305-719, South Korea

**Keywords:** dewetting, metal thin films, nanoimprint, nanoparticles, self-assembly

## Abstract

We propose a nanofabrication process to generate large-area arrays of noble metal nanoparticles on glass substrates via nanoimprinting and dewetting of metallic thin films. Glass templates were made via pattern transfer from a topographic Si mold to an inorganically cross-linked sol–gel (IGSG) resist on glass using a two-layer polydimethylsiloxane (PDMS) stamp followed by annealing, which turned the imprinted resist into pure silica. The transparent, topographic glass successfully templated the assembly of Au and Ag nanoparticle arrays via thin-film deposition and dewetting at elevated temperatures. The microstructural and mechanical characteristics that developed during the processes were discussed. The results are promising for low-cost mass fabrication of devices for several photonic applications.

## Findings

Thin films on nonreactive solid surfaces, having a high surface area relative to their volume, are energetically metastable and can dewet or agglomerate into particles when exposed to a high temperature. Substrates with periodic topography can be used to direct or control the dewetting process to form ordered arrays of nanoparticles governed by the topographic features of the underlying surface [1–2]. Because it is a relatively simple process [3], this technique opens up numerous applications, such as high-density magnetic recording media [2,4], photovoltaic devices [5–10], photocatalysts [11] and catalysts for the fabrication of carbon nanotubes and nanowires. However, high-resolution lithography techniques – such as electron beam lithography (EBL) or laser interference lithography (LIL) – with a conventional multistep etching process on a silicon wafer are still required to fabricate templates with nanostructured surface topographies that determine the features of the dewetted nanoparticles [1,12–15]. These complicated processing steps for prepatterned templates critically restrict extensive application of the thin-film dewetting technique. Furthermore, lithography techniques based on silicon wafers have limitations when the template must also serve as a functional layer. Thus, the applicability of a self-assembly technique that uses dewetting largely depends on how it can be combined with appropriate template materials that have both functionality and dewettability.

In this regard, nanoimprint lithography (NIL) is expected to be an effective substitute in those processes for the fabrication of topographic surfaces. For easier and faster processing, the imprinted resists should be directly used as a functional layer. Therefore, they must have adequate thermal and mechanical stability without undergoing relaxation during the dewetting of the metallic thin films at high temperatures. Hybrid sol–gel thin films prepared from methyltriethoxysilane (MTEOS) may fulfill these requirements due to the complete hydrolyzation of the ethoxy groups and decomposition of the methyl groups [16–17]. MTEOS films have attracted attention as self-sustainable films for gas-selective membranes and due to their superhydrophobic and low-dielectric properties [18–21]. The films are also applicable in optics and photonics because they can interact with light and actively induce changes in their physical properties [22–23].

There is increasing demand for nanoparticles deposited on glass for various optical or photonic devices [24–25]. Well-ordered particle arrays on a glass substrate can change its optical response or absorbance spectra [26–30]. This article aims to demonstrate a method for sequentially fabricating arrays of Au and Ag nanoparticles on glass substrates using NIL with transparent MTEOS film and dewetting techniques that can be applied for the photonic devices with sufficient structural integrity.

A master mold of square arrays of inverted pyramidal pits with a period of 200 nm was made from (100) silicon wafers with a 40 nm thick silicon nitride layer using a laser interference lithographic (LIL) method. The topography of the master mold was replicated on a composite stamp consisting of two layers – hard polydimethylsiloxane (PDMS) and flexible 184 PDMS [31]. A 50 µm thick layer of hard PDMS and 2 mm thick layer of 184 PDMS were supported on a 1 mm thick glass substrate. The layer thicknesses in the two-layer stamps were determined in order to minimize the distortion of the hard PDMS layer that can occur due to the difference between the stiffness and thermal expansion coefficients of hard PDMS and those of the soft 184 PDMS.

In the next imprinting step, an inorganically cross-linked sol–gel (ICSG) resist with a thickness (*t*_f_) of 400 nm was spin-coated on a Pyrex glass substrate. To improve the wettability of the resist, the glass surface was pretreated with a dielectric barrier discharge (DBD) using He and O_2_ plasmas. To condense the silanol groups (Si–OH) in the resist into siloxane bonds (Si–O–Si) and form cross-linked networks, the resist was heated to 110 °C for different times while pressing with the stamp. Thereafter, the resists with inverted pyramidal pits were heated to different annealing temperatures to completely transform them into pure silica by oxidizing and decomposing the CH_3_ groups in the resist. The maximum annealing temperature was limited to 600 °C, up to which the soda lime glass does not undergo deformation. Noble metals (Au and Ag) were sputter-deposited onto the imprinted resist with the periodic array of inverted pyramidal pits and annealed in a furnace at ≈300–500 °C to assemble nanoparticle arrays via solid-state dewetting of the deposited films. The base pressure and RF power of the sputtering system were 3 × 10^−6^ Torr and 100 W, respectively.

Fourier transform infrared (FTIR) spectral analysis was conducted on the resists just after pressing at 110 °C for different times using an FTIR spectrometer (Nicolet 6700) over the 2400–600 cm^−1^ spectral range at room temperature. To examine the mechanical stability of the ICSG resists after annealing at different temperatures, nanoindentation measurements were conducted. The measurements were performed using a nanoindentation system (MTS Nano-indenter XP) equipped with continuous stiffness measurement using a Berkovich indenter, and the elastic modulus and nanohardness were calculated using the method of Oliver and Pharr [32]. The morphologies of the stamp, resists with topography and assembled nanoparticles were examined using scanning electron microscopy (SEM).

**Figure 1:**
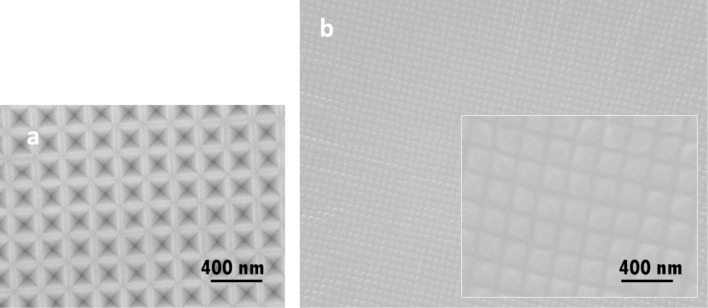
(a) Silicon master mold with inverted pyramidal pits and (b) PDMS stamp with the transferred pattern. The inset in (b) is a high-magnification image.

**Figure 2:**
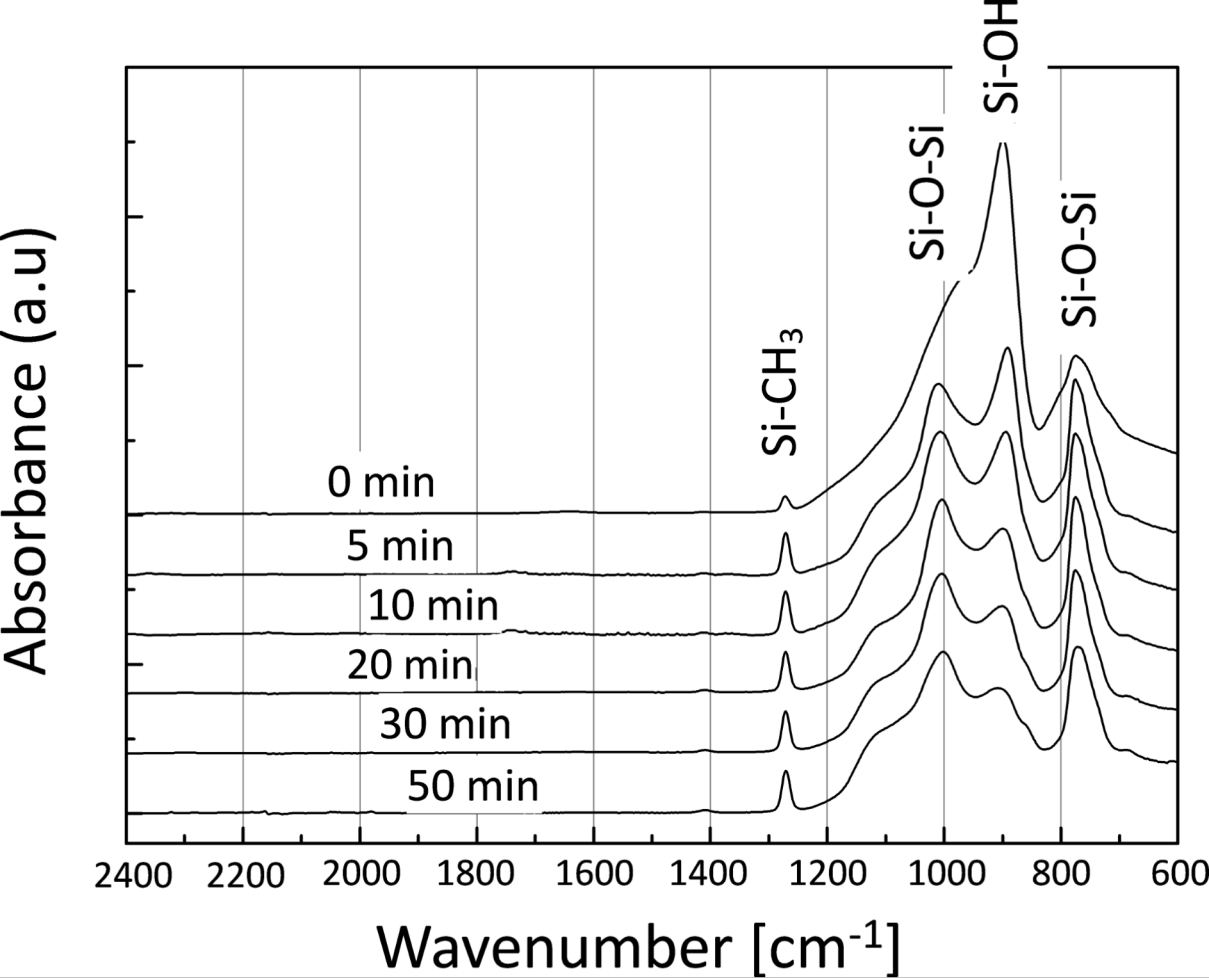
Evolution of the FTIR absorption spectra of the ICSG resists during condensation at 110 °C for different times.

Figure 1 shows the surface appearances of the silicon master mold with inverted pyramidal pits and the stamp replicating the mold topography. Figure 2 shows the changes in the FTIR transmission spectra with increasing condensation time. All spectra, except that corresponding to the nonannealed sample, were normalized to the CH_3_ deformation band at 1267 cm^−1^. As the annealing time increased, the silanol absorption peak at 895 cm^−1^ decreased, while the siloxane stretching resonances at 780 and 1020 cm^−1^ increased. According to Peroz et al. [16], when the intensity ratio (τ_SiOH_) at *t* min to 0 min is <0.3, the patterns are stable upon subsequent high-temperature annealing. Figure 2 indicates that this occurs after more than 30 min of annealing in our experiment.

**Figure 3:**
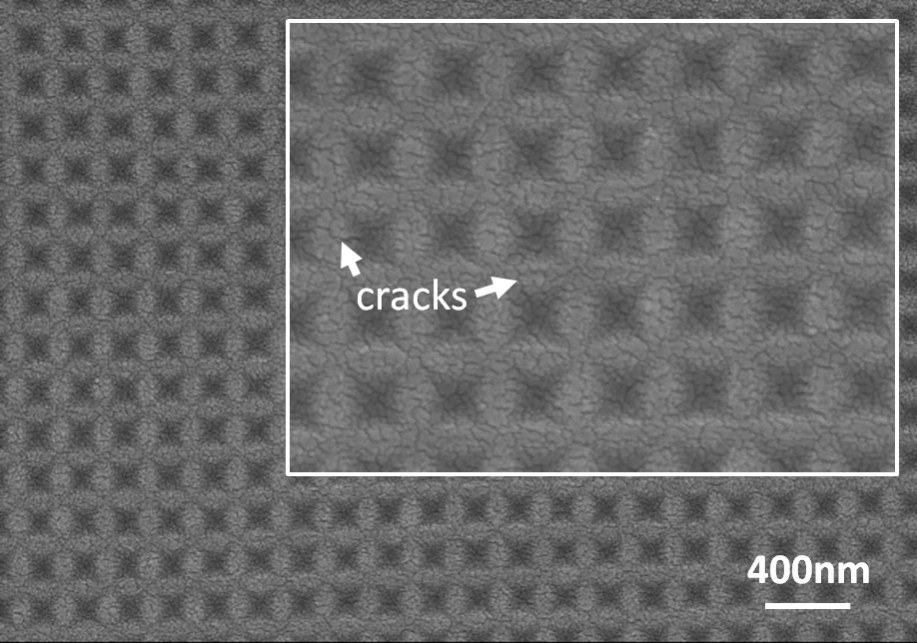
SEM images showing the surface topography of the ICSG resist after annealing at 550 °C for 5 h. The inset image shows small surface cracks in the resist at higher magnification.

The resists imprinted at 110 °C for 30 min were annealed in the temperature range of ≈400–600 °C to transform them into pure silica. Figure 3 shows an example of the imprinted surface features after annealing at 550 °C for 5 h. The topography of the master mold was mostly transferred onto the silica on glass, although there was some smoothing of the inverted pyramidal edges and corners. There was no modification of the pattern amplitude despite the shrinking during the transformation to silica. However, small cracks inevitably formed on the surface due to densification during the transformation (Figure 3, inset)

**Figure 4:**
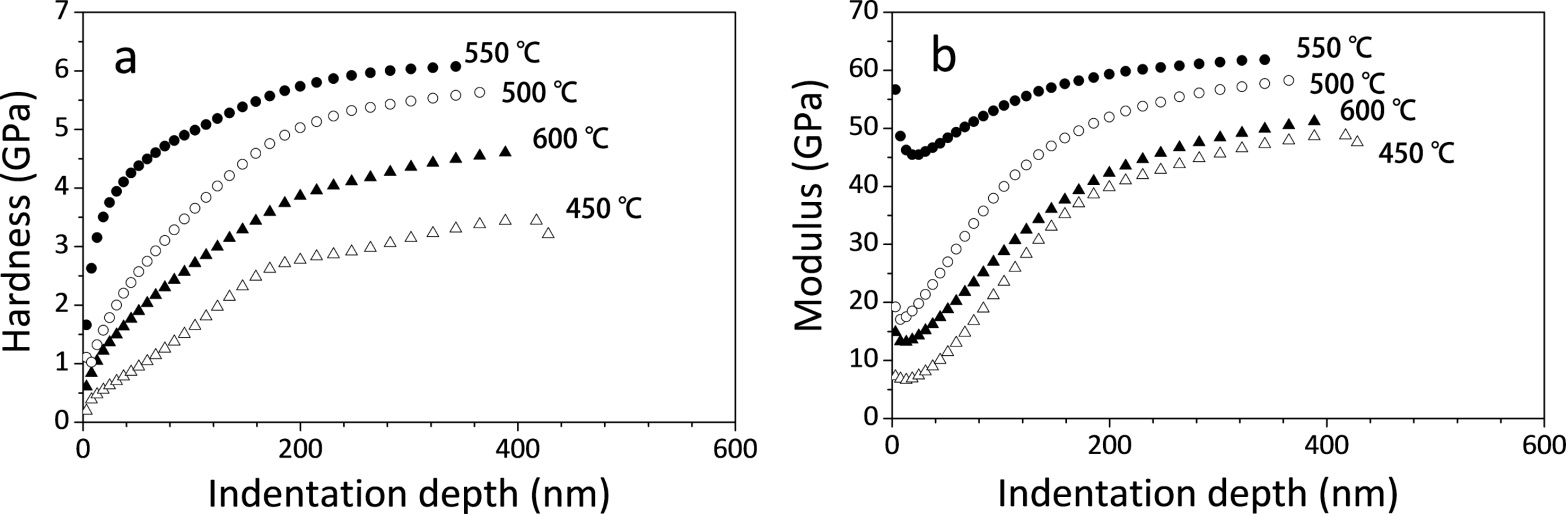
Nanoindentation measurement for the ICSG resists annealed at different temperatures: (a) hardness and (b) elastic modulus.

To examine the effect of the annealing temperatures on the mechanical stability of the structure, nanoindentation measurements were conducted for the resists on glass annealed at ≈400–600 °C. Figure 4a,b shows the changes in the composite hardness and elastic modulus of the thin resist–glass substrate as a function of the indentation depth (*h*). The Young’s modulus rapidly decreased as the indentation depth increased to ≈20 nm. In the nanoindentation measurements, the Young's modulus (*E*_r_) is given by *E*_r_ = (√π/2β)(d*P*/d*h*)/√*A* where β is a constant, (d*P*/d*h*) is the slope of the load–displacement curve at the beginning of the unloading stage, and *A* is the projected area of the contact [33]. The surface conditions, such as the presence of small cracks or open pores on the resist, may affect (d*P*/d*h*) and sharply decrease the modulus at such small depths. The rounded tip of the Berkovich indenter (≈50 nm) may also sharply increase the contact area (*A*) at the beginning of indentation and decrease the modulus [33]. Nonetheless, both the modulus and the hardness of all annealed resists, with the exception of the modulus behavior at extremely small indentation depths, increased as the indentation depth increased. This result strongly supports the effect of a stiffer substrate on the properties of thin films, as already indicated by other researchers [33–34]. However, despite this effect, the plots clearly reveal the effect of the annealing temperature on the mechanical properties of the resists: the modulus and hardness increased with increasing annealing temperature, reaching the maximum at 550 °C. The composite hardness and modulus of the resist–glass system annealed at 550 °C were 5.6 and 58 GPa, respectively, at the indentation depth of *t*_f_/2. Considering the properties of the Pyrex glass substrate, which has a hardness of 7 GPa and a modulus of 55 GPa, the resist was expected to mostly transform into pure silica in the annealing process at 550 °C. The resist annealed at 600 °C showed dramatic degradation of its mechanical properties. This is attributed to the residual stress built up between the converted silica and the substrate glass, which was heated above its glass transition temperature (564 °C) during annealing. Indeed, some glass substrates were observed to be slightly deformed after annealing at 600 °C.

**Figure 5:**
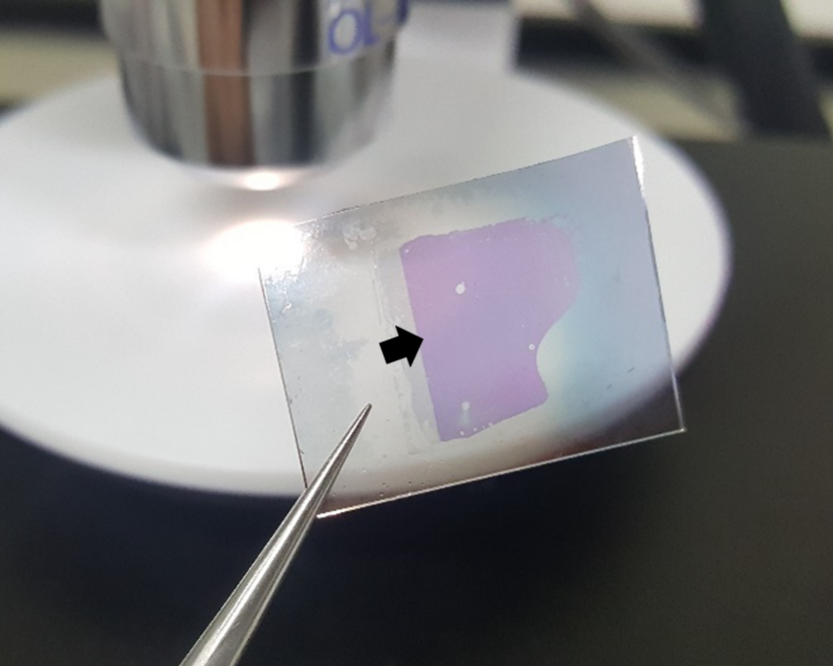
Photograph of transparent glass with Ag nanoparticle arrays.

**Figure 6:**
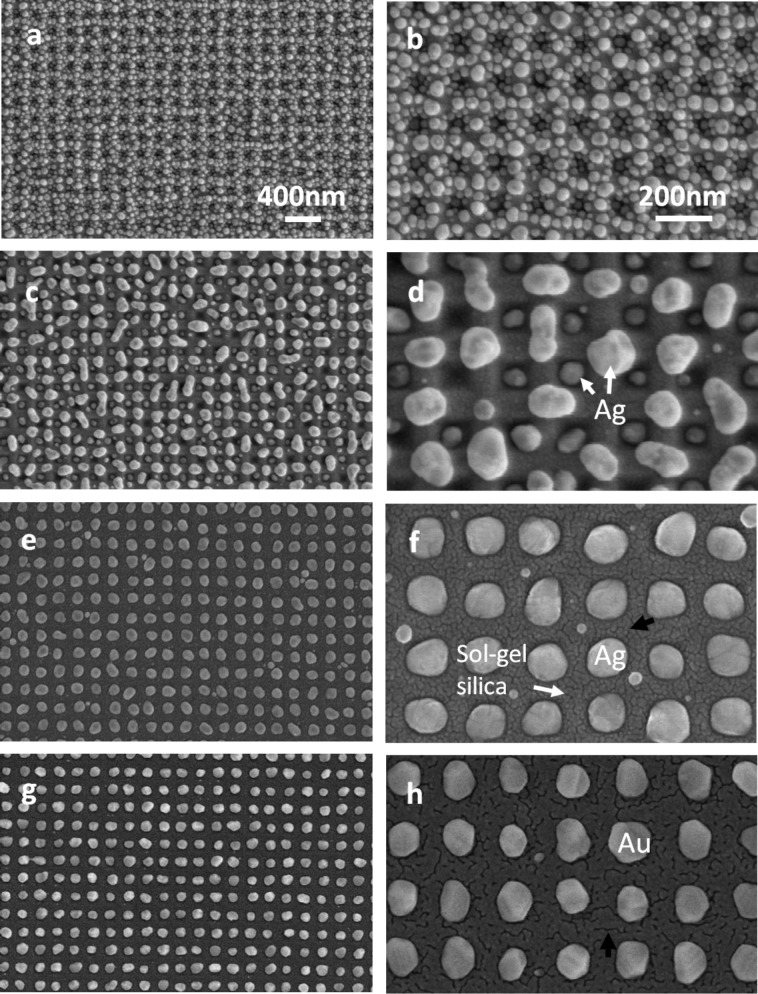
Metal nanoparticles formed on imprinted sol–gel silica: (a,b) 8 nm thick and (c,d) 10 nm thick Ag films dewetted at 300 °C, (e,f) 14 nm thick Ag and (g,h) Au films dewetted at 500 °C. Black arrows in images f and h indicate surface cracks formed in imprinted sol–gel silica.

Thin films of noble metals were deposited on imprinted resists that were converted to sol–gel silica at 550 °C and then dewetted at high temperatures (≈300–500 °C) to form nanoparticles. Figure 5 shows the visual appearance of a transparent glass substrate with a Ag nanoparticle array at low magnification. The metal nanoparticles formed in a square region of approximately 20 × 20 mm^2^ on the glass (arrowed). Figure 6 shows SEM images of the regions with Ag and Au nanoparticles. The initial film thickness and dewetting temperatures determined the shape and distribution of the dewetted particles on glass. In Figure 6a,b, small nanoparticles clustered around the pits as an 8 nm thick Ag film was dewetted at 300 °C. In Figure 6c,d, one can see that an ordered array of small particles formed in the pits, while large agglomerates formed on the mesa during the dewetting of the 10 nm thick Ag film. Well-ordered arrays of Ag particles appeared when ≈12–15 nm films were annealed at ≈400–500 °C, forming an assembly of one metal nanoparticle per pit in the topographic patterns in the silica template, as shown in Figure 6e. This was the same for the Au films (Figure 6g). This evolution of the particle assembly with film thickness and dewetting temperature is very similar to previous works performed on silicon templates (or master molds) with the same inverted pyramidal pits [2,10,12], demonstrating that the fabrication of topographic templates via the imprinting method can successfully replace LIL on silicon wafers, which is necessary for the assembly of ordered nanoparticles via solid-state dewetting.

**Figure 7:**
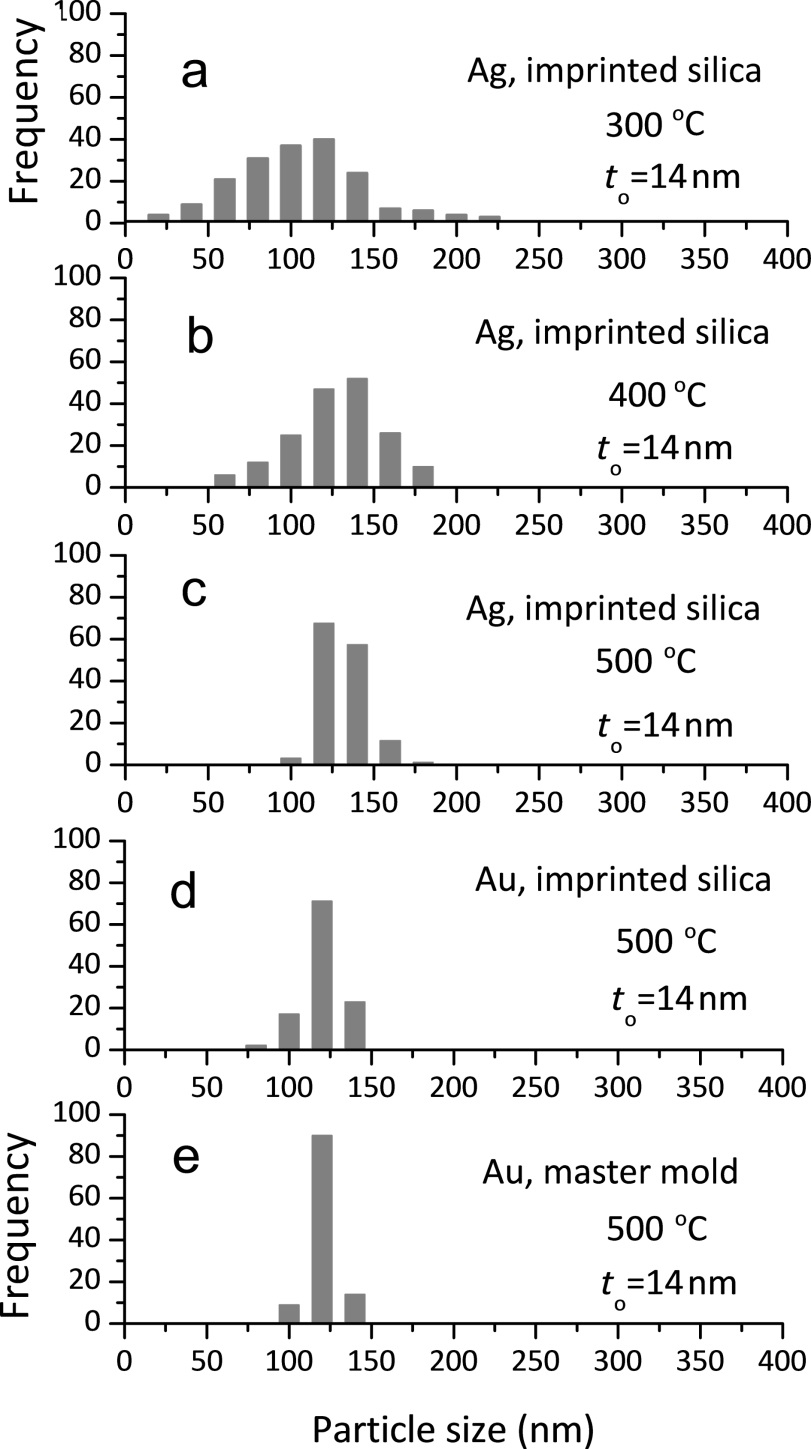
Size distribution of (a–c) Ag and (d) Au particles formed at different dewetting temperatures on imprinted sol–gel silica, and (e) Au particles formed on the silicon master mold.

The dewetting processes of polycrystalline metallic films on smooth and topographic substrates are well known. On smooth substrates, solid-state dewetting of the film occurs with film thinning and hole formation at a grain boundary (GB) triple junction, followed by retraction of void edges due to capillary-driven diffusion, and is completed with the formation of particles [35–36]. On a silicon template with inverted pyramidal pits (lightly oxidized to form 10 nm thick silica), however, the pits in the template modulate in the curvature of the as-deposited film, which induces grooving and breakage of the film at the edges of the pits (or mesas) with high curvature before the film breaks at GB triple junctions [1,12–13]. This mechanism enables the formation of a single isolated particle inside the pit. If the as-deposited polycrystalline film is so thin that it has high surface roughness relative to the film thickness or is not continuous, the film will break up into multiple fine particles clustered around the pits and mesas. This is the case of Figure 6a,b for the 8 nm thick Ag film on the imprinted sol–gel silica. The size uniformity of the assembled metal particles can increase with increasing annealing temperature. This is attributable to the faster dewetting kinetics at higher temperatures, which induces rapid breakage of the film along the mesa with high surface curvature and fast retraction of the film edges, preventing both film thinning at GB triple junctions and the agglomeration of the film across the pits. Figure 7a–c shows the size distribution of NPs dewetted at different temperatures for a 14 nm thick Ag film. With increasing dewetting temperature, the plots became narrower, eliminating fine and coarse particles. At 500 °C, the plot showed the strongest size uniformity (Figure 7c) because most particles were assembled into the ordered array of the pits in the template as shown in Figure 6e.

**Figure 8:**
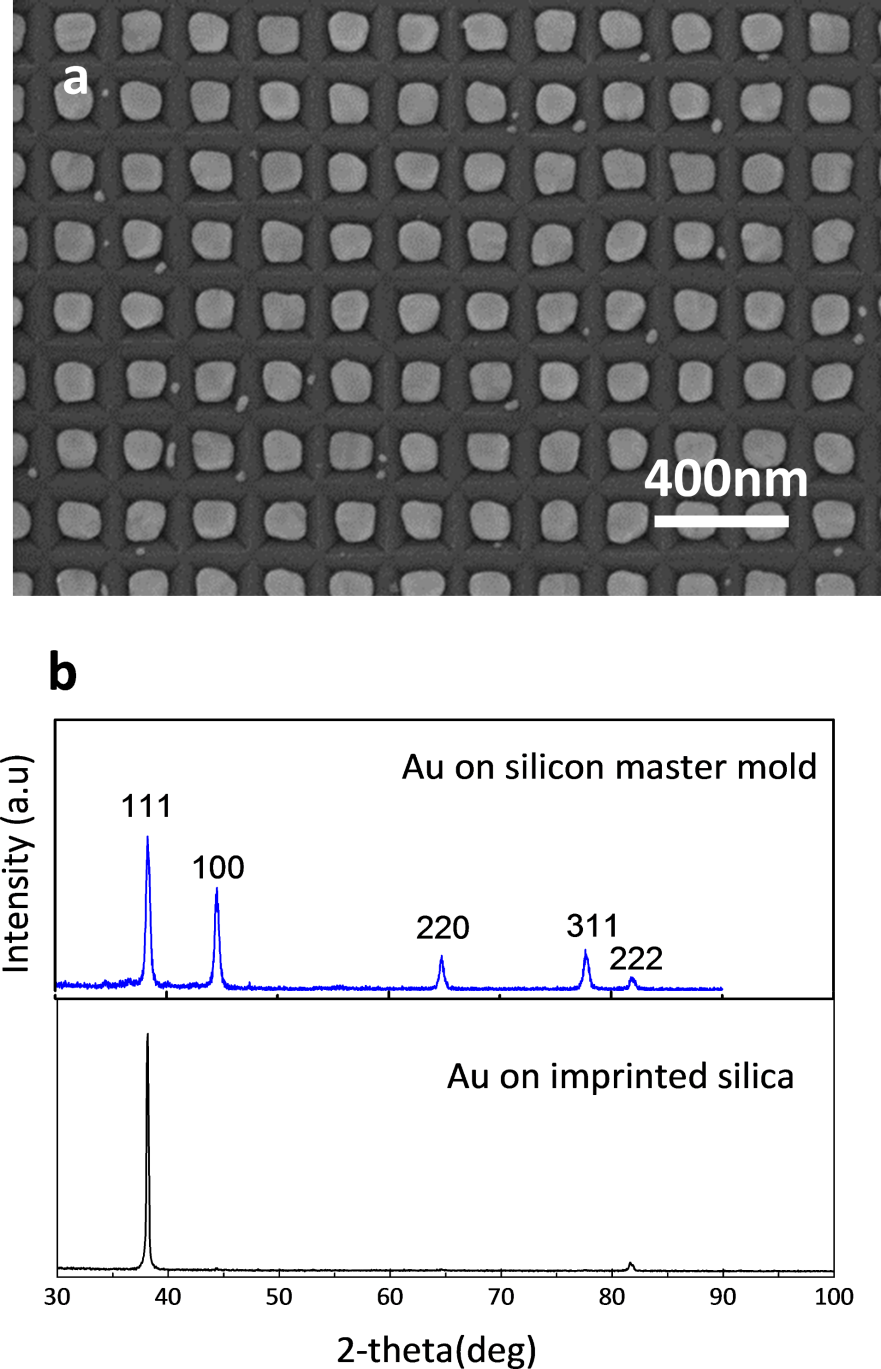
(a) SEM image of Au nanoparticles dewetted on the silicon master mold by annealing at 500 °C and (b) a comparison of XRD spectra for Au particle arrays formed on the imprinted sol–gel silica in Figure 6h and silicon master mold in Figure 8a.

Figure 6f and Figure 6h show many small cracks on the silica substrate. However, despite these defects, due to the highly hydrophobic properties of the imprinted silica, the Ag and Au films were successfully dewetted into the pits and formed uniform nanoparticles with only small residuals on top of the silica surface. The dewetted Au particles directly formed on the Si master molds by annealing at 500 °C; their appearance and size distributions are shown in Figure 8 and Figure 7e together with data for the Au particles formed on the imprinted sol–gel silica in Figure 7d. Comparing the plots shown in Figure 7e and Figure 7d, thin-film dewetting using the imprinted silica (Figure 7d) resulted in only a slight loss of size uniformity and periodicity. However, the XRD patterns in Figure 8b show that the particles assembled on the Si master mold and imprinted silica templates have different crystal orientations: (111) and (100) are the preferred orientations on the master mold, while only (111) orientation is preferred on the imprinted silica. According to a previous report [11], the (100) orientation of Au particles on a Si template with inverted pyramidal pits predominates when the cubic (111) crystal planes, which have the lowest surface energy, become parallel to the facets of the pits. Therefore, we assume that the imprinted facets of the pits on the sol–gel silica were partly relieved or surface cracks on the converted silica disturbed the development of (111) planes parallel to the facets of the pits. Nevertheless, for the mass production of metal nanoparticle arrays, using the sol–gel silica template, which replicates the topography of the silicon master mold, can be a useful and cost-effective process that substitutes for the use of a silicon template produced using the LIL method.

In conclusion, ordered arrays of noble metal nanoparticles were assembled on glass templates using a combination of nanoimprinting and thermal dewetting of metallic thin films in a controllable manner. The imprinted topography on sol–gel silica successfully guided the solid-state dewetting process of noble metal films at high temperature without structural degradation. By providing a simple route to assemble nanoparticles on inexpensive large-area substrates, the method presented here can extend the applications of metal nanoparticles for several optical purposes.
